# Hepatic angiomyolipoma, misdiagnosed as hepatocellular carcinoma

**DOI:** 10.1093/jscr/rjad556

**Published:** 2023-10-14

**Authors:** Bárbara M Marinho, António G Canha, Donzília S Silva, Ana P Rodrigues

**Affiliations:** General Surgery Service, Department of Surgery, Centro Hospitalar Universitário do Porto, EPE, 4099-001 Porto, Portugal; Hepatobiliary and Pancreatic Unit, Department of Surgery, Centro Hospitalar Universitário do Porto, EPE, 4099-001 Porto, Portugal; Hepatobiliary and Pancreatic Unit, Department of Surgery, Centro Hospitalar Universitário do Porto, EPE, 4099-001 Porto, Portugal; Anatomical Pathology Service, Department of Pathology, Centro Hospitalar Universitário do Porto, EPE, 4099-001 Porto, Portugal

## Abstract

Perivascular epithelioid cell neoplasm (PEComa) is a rare type of tumor, and hepatic PEComa is even rarer. Its preoperative diagnosis is difficult, given the absence of specific clinical manifestations, often constituting an accidental finding, and the lack of a gold standard for identification using imaging studies. Instead, the diagnosis of hepatic PEComa is based on morphological and immunohistochemical features. We describe a case of an asymptomatic hepatic PEComa, angiomyolipoma type, which appeared in a middle-aged woman with chronic liver disease, during her follow-up and screening. Given the patient's context, human immunodeficiency virus-positive with chronic hepatitis C, and the similarities between the two tumors, the hepatic lesion was interpreted as hepatocellular carcinoma. The patient underwent surgical excision of the tumor, and the positive immunohistochemical staining for human melanoma black 45 and Melan A made the definitive diagnosis. In the absence of aggressiveness tumor markers, surveillance was decided. We also provide a literature review of these tumors.

## Introduction

Angiomyolipomas (AML) are rare mesenchymal tumors belonging to a group of perivascular epithelioid cell tumors (PEComas), most commonly encountered in the kidney. The liver is the second most common site of AML occurrence [1, 2] and 6%–10% of the patients with hepatic AMLs (HAMLs) suffer from tuberous sclerosis [3]. PEComas show a wide anatomical distribution but hepatic PEComas are very rare [4, 5].

The tumor is characterized by its composition with blood vessel, smooth muscle and adipose tissue of varying proportions. This particular tissue composition of HAML makes it hard to distinguish from other liver tumors [1]. They can present similarly to malignant tumors, such as hepatocellular carcinoma, both clinically and radiologically [6]. Correct preoperative diagnosis of HAML is reported to be <25% [3].

Conventional imaging examinations are the major preoperative diagnostic tools. HAMLs are usually soft, solitary, well encapsulated tumors, and typically have heterogeneous enhancement on both arterial and venous phases [4, 7]. However, HAMLs are difficult to diagnose radiologically, because of the variability of their growth patterns, various proportions of the tissue components, and their proximity to major hepatic vascular structures. Therefore, misdiagnosis of HAML remains high [6].

Histopathological examination along with the distinctive immunohistochemical positivity for human melanoma black 45 (HMB45) and smooth muscle markers, are confirmative for HAML [8, 9].

Although malignant behavior is scarcely seen, the treatment is mostly surgical. However, a conservative approach may be appropriate in the face of a definitive diagnosis of AML and absence of severity criteria [6, 10].

## Case report

We present a clinical case of a woman in her 40s with human immunodeficiency virus infection on antiretroviral therapy (ART), treated HCV co-infection with chronic liver disease in this context, smoker, and with past medical history of heroin use, pneumocystosis, lymph node tuberculosis and syphilis. In addition to ART, the patient was medicated with sertraline for refractory depression after her father's death. Her father had recently died of hepatocellular carcinoma (HCC), the only relevant family history.

**Figure 1:**
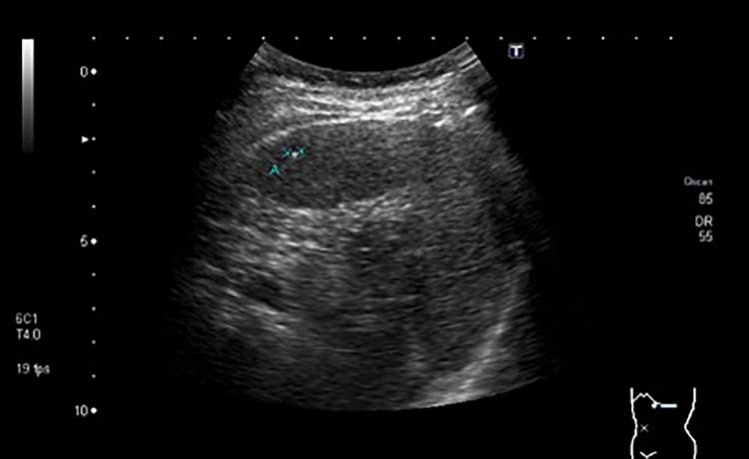
Ultrasound showing a hypoechoic nodule, vaguely heterogeneous, located in segment VII, subcapsular, and with ~22 mm in diameter.

**Figure 2:**
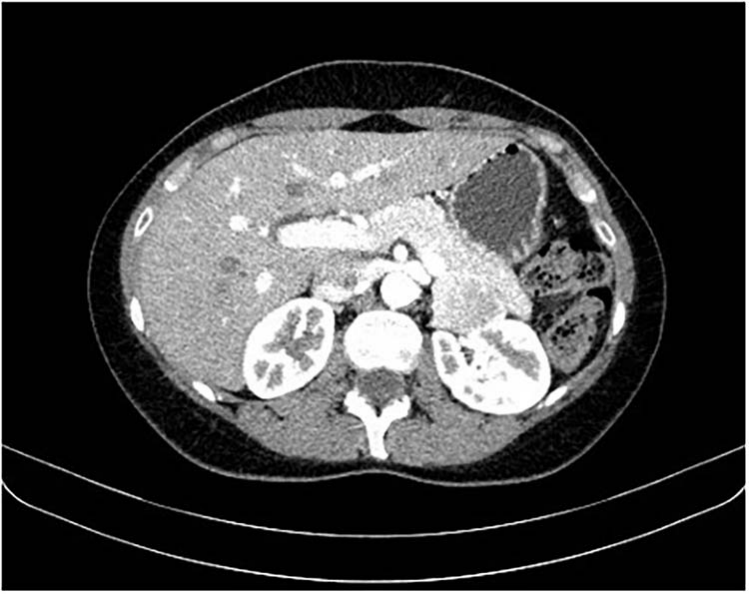
CT scan showing, in addition to signs of chronic liver disease, the presence of a solid subcapsular nodule of segment VII, measuring 23 mm, with hypervascular behavior in the arterial phase.

**Figure 3:**
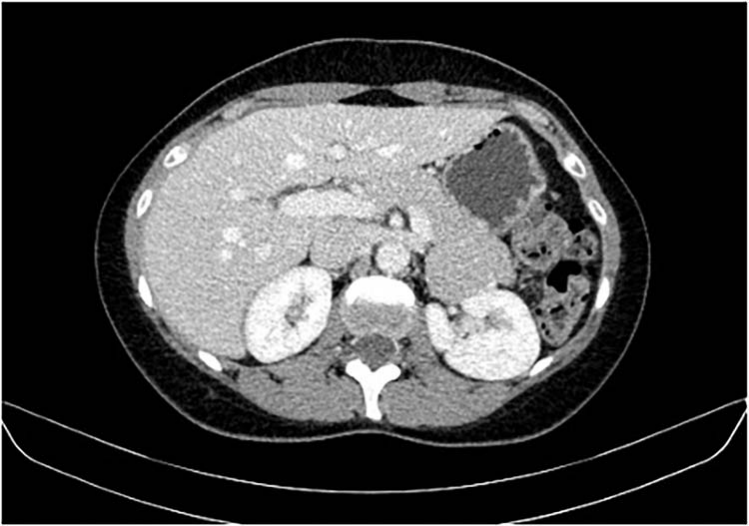
CT scan showing the same mass as shown in Fig. 2 but with washout in the portal venous phase.

During a screening imaging examination of her liver disease, a lesion suspected of malignancy was detected. The ultrasound disclosed a hypoechoic nodule, vaguely heterogeneous, located in segment VII, subcapsular, and with ~22 mm in diameter (Fig. 1). Liver blood tests and serum alpha-fetoprotein level were normal. For better characterization, a CT scan was performed. The CT showed, in addition to signs of chronic liver disease, the presence of a solid subcapsular nodule of segment VII, measuring 23 mm, with hypervascular behavior in the arterial phase (Fig. 2) and washout in late venous phase (Fig. 3).

Based on the imaging characteristics and the patient's history, hepatocellular carcinoma was assumed and its surgical excision was proposed. The patient underwent intraoperative ultrasound and laparoscopic resection of the liver lesion, with macroscopic margins of ~1 cm. The procedure and the postoperative period were uneventful.

**Figure 4:**
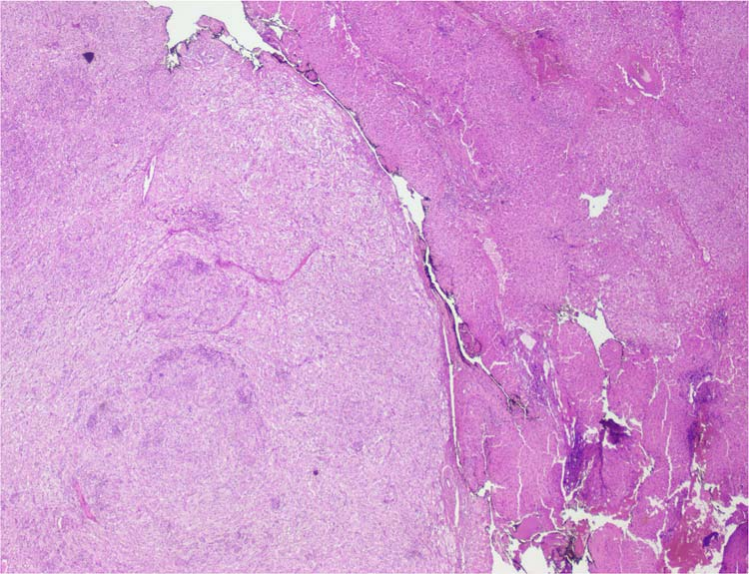
Liver parenchyma partially occupied by a neoplastic proliferation with expansive borders.

**Figure 5:**
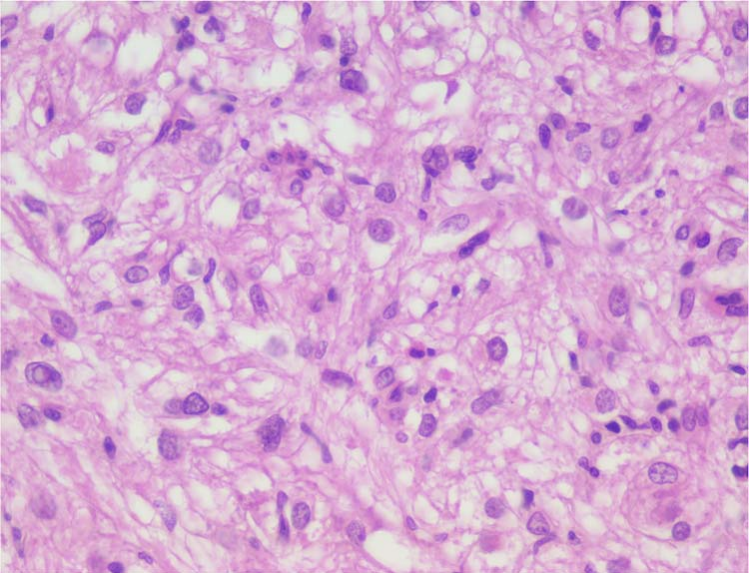
The neoplastic cells are organized in small bundles. The cells are spindled to oval, with eosinophilic and granular cytoplasm and with mild nuclear atypia.

**Figure 6:**
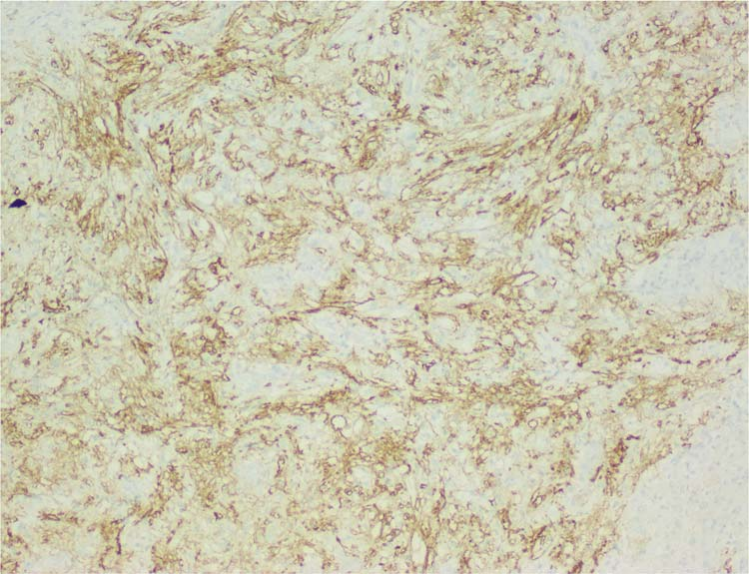
There is heterogeneous positivity for smooth muscle markers.

**Figure 7:**
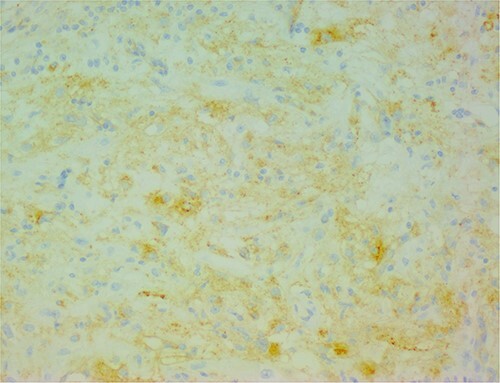
The neoplastic cells are also positive for HMB45, demonstrating the typical myo-melanocytic differentiation.

The histological evaluation of the surgical specimen revealed a storiform fusiform cell neoplasm with expansive borders, areas of a myopericytomatous pattern, and the presence of rhabdoid cells (Fig. 4). The neoplastic cells, spindled to oval, with eosinophilic and granular cytoplasm and with mild nuclear atypia, were organized in small bundles (Fig. 5). Anomalous arterial structures were observed. Neither necrosis nor mitotic figures were identified. The immunohistochemical study revealed immunoreactivity of neoplastic cells for SMA, HHF35 (Fig. 6) and HMB45 (Fig. 7), Calponin, S100 and CD31. The margins did not intersect the neoplasm. Thus, contrary to the preoperative diagnosis of hepatocellular carcinoma, the morphological aspects and the immunohistochemical profile favored the diagnosis of epithelioid angiomyolipoma.

Given the absence of characteristics suggestive of aggressiveness and once the surgical resection was complete, surveillance was proposed. There was no evidence of recurrence during the first year of follow-up.

## Discussion

In 2013, the World Health Organization defined PEComas as mesenchymal tumors composed of distinctive cells that show a focal association with blood vessel walls, and usually express melanocytic and smooth-muscle markers [8]. Its etiology remains uncertain; however, many hypotheses exist regarding the cell of origin [4]. HAMLs, a subset of PEComas, are extremely rare, have a higher incidence in women, and are usually found in non-cirrhotic livers with an unclear pathogenesis [2]. The majority seem to occur in adult females of Asian countries [1].

Clinical manifestations of PEComa in general, including hepatic PEComa, are uncommon [5]. As in our case, most patients are asymptomatic or have nonspecific symptoms, and the tumors are found incidentally on imaging studies performed for other reasons. Although commonly asymptomatic, presentation can range from a palpable abdominal mass or vague pain to acute abdomen [4]. In large subcapsular tumors, rupture with hemoperitoneum may occur [8, 9].

The natural history of primary hepatic PEComas is quite varied and not yet well established [4]. They are primarily benign tumors, but aggressive PEComas with malignant potential and metastatic risk have been reported [5]. Malignant transformation is more likely in the presence of high-risk features [3, 4].

In accordance with clinical presentations, there are no specific imagiological signs [7]. Regardless of their site of occurrence, AMLs are generally composed of epithelioid and spindle myoid cells, with a clear to pale granular cytoplasm, mature adipose tissue, and sinusoid-like vessels, in varying proportions [4, 6]. Immunostaining characteristics are consistent with melanocytic and smooth muscle differentiation [8, 9]. HMB45 is considered the most sensitive marker for PEComa, followed by Melan-A. Smooth muscle actin (SMA) is the most widely described smooth muscle marker [11].

In this report, we present a rare case of hepatic epithelioid AML in a 45-year-old woman. Our patient presented with an asymptomatic liver mass, incidentally detected on a screening examination due to her chronic hepatitis C, which is considered to be unrelated to PEComas. They have been found mostly in livers without a background of cirrhosis or hepatitis. [4] The tumor demonstrated the characteristic pathologic features of AML, such as the presence of spindle myoid cells, mature adipose tissue and sinusoid-like vessels. Epithelioid cells were the predominant cell type, making the differentiation from hepatocellular carcinoma challenging. Immunohistochemistry was pathognomonic for AML, as epithelioid and spindle myoid cells were positive for melanocytic (HMB45) and myogenic (SMA) markers.

Radical resection remains the mainstay of PEComa treatment because these tumors show a high resistance to radiotherapy and chemotherapy [10], and, usually, there is no recurrence after initial resection [4]. Malignant behavior of HAML with invasive growth, recurrence after surgical resection and even metastasis has been described in ~4% of patients [2]. Conservative management may be suitable in asymptomatic patients with small lesions, without any high-risk features [6]. Our patient, given the preoperative diagnostic suspicion of HCC, underwent laparoscopic resection.

## Data Availability

The authors confirm that all data underlying the results are available within the article and its supplementary materials. No additional source data are required.

## References

[1] Ding GH, Liu Y, Wu MC, Yang GS, Yang JM, Cong WM. Diagnosis and treatment of hepatic angiomyolipoma. J Surg Oncol 2011;103:807–12. 21283992 10.1002/jso.21814

[2] Klompenhouwer AJ, Dwarkasing RS, Doukas M, Pellegrino S, Vilgrain V, Paradis V. et al. Hepatic angiomyolipoma: an international multicenter analysis on diagnosis, management and outcome. Hpb [Internet] 2020;22:622–9. 31619346 10.1016/j.hpb.2019.09.004

[3] Attard A, Piscopo N, Schembri J, Buhagiar T, Cortis K, Ellul P. A rare case of PEComa of the liver. GE Port J Gastroenterol 2021;28:217–21. 34056048 10.1159/000509192 PMC8138188

[4] Khaja F, Carilli A, Baidas S, Sriharan A, Norford S. PEComa: a perivascular epithelioid cell tumor in the liver - a case report and review of the literature. Case Rep Med 2013;2013:4–8. 10.1155/2013/904126 PMC3891746 24489554

[5] Khan HM, Katz SC, Libbey NP, Somasundar PS. Hepatic PEComa: a potential pitfall in the evaluation of hepatic neoplasms. BMJ Case Rep 2014;2014:bcr2014204122–5. 10.1136/bcr-2014-204122 PMC4054585 24907216

[6] Damaskos C, Garmpis N, Garmpi A, Nonni A, Sakellariou S, Margonis GA. et al. Angiomyolipoma of the liver: a rare benign tumor treated with a laparoscopic approach for the first time. In Vivo (Brooklyn) 2017;31:1169–73. 10.21873/invivo.11185 PMC5756647 29102941

[7] Chen Z, Han S, Wu J, Xiong M, Huang Y, Chen J. et al. A systematic review: perivascular epithelioid cell tumor of gastrointestinal tract. Medicine (Baltimore) 2016;95:e3890. 27428182 10.1097/MD.0000000000003890 PMC4956776

[8] Son HJ, Kang DW, Kim JH, Han HY, Lee MK. Hepatic perivascular epithelioid cell tumor (PEComa): a case report with a review of literatures. Clin Mol Hepatol 2017;23:80–6. 28288506 10.3350/cmh.2016.0034 PMC5381835

[9] Shakuntala PN, Shilpashree M, Geethanjali S, Sharma SK. Acute abdomen as an unusual presentation of broad ligament angiomyolipoma. A case report and review of literature. Indian J Surg Oncol 2012;3:276–8. 24293961 10.1007/s13193-012-0169-8 PMC3521548

[10] Sobiborowicz A, Czarnecka AM, Szumera-Ciećkiewicz A, Rutkowski P, Świtaj T. Diagnosis and treatment of malignant PEComa tumours. Oncol Clin Pract 2020;16:22–33.

[11] Girardi FM, Nunes AB, Hauth LA. Malignant subcutaneous PEComa on the cheek. An Bras Dermatol 2018;93:934–5. 30484551 10.1590/abd1806-4841.20187595 PMC6256217

